# Single-cell barcode analysis provides a rapid readout of cellular signaling pathways in clinical specimens

**DOI:** 10.1038/s41467-018-07002-6

**Published:** 2018-10-31

**Authors:** Randy J. Giedt, Divya Pathania, Jonathan C. T. Carlson, Philip J. McFarland, Andres Fernandez del Castillo, Dejan Juric, Ralph Weissleder

**Affiliations:** 1 Center for Systems Biology, Massachusetts General Hospital, 185 Cambridge St, CPZN 5206, Boston, MA 02114 USA; 2 MGH Cancer Center, Massachusetts General Hospital, 185 Cambridge St, CPZN 5206, Boston, MA 02114 USA; 3 Department of Systems Biology, Harvard Medical School, 200 Longwood Ave, Boston, MA 02115 USA

## Abstract

Serial tissue sampling has become essential in guiding modern targeted and personalized cancer treatments. An alternative to image guided core biopsies are fine needle aspirates (FNA) that yield cells rather than tissues but are much better tolerated and have lower complication rates. The efficient pathway analysis of such cells in the clinic has been difficult, time consuming and costly. Here we develop an antibody-DNA barcoding approach where harvested cells can be rapidly re-stained through the use of custom designed oligonucleotide-fluorophore conjugates. We show that this approach can be used to interrogate drug-relevant pathways in scant clinical samples. Using the PI3K/PTEN/CDK4/6 pathways in breast cancer as an example, we demonstrate how analysis can be performed in tandem with trial enrollment and can evaluate downstream signaling following therapeutic inhibition. This approach should allow more widespread use of scant single cell material in clinical samples.

## Abstract (web summary)

Diagnostic tests need optimization to avoid invasive and costly repeated biopsies. Here the authors present an antibody-DNA barcoding approach where harvested single cells can be re-stained through custom oligonucleotide-fluorophore conjugates, enabling multiplexed analysis of cancer pathways.

## Introduction

Modern oncology increasingly relies on pathological, molecular, and genomic assessments of biopsied tumor tissue to guide treatment selection and to evaluate therapeutic response or resistance. There are also other reasons for sampling tumors frequently beyond the initial biopsy to establish a diagnosis: (i) the realization that tumors can adapt rapidly to therapeutic pressures causing resistance, (ii) the emergence of many novel targeted therapies and nanotechnologies efficacious only in subsets of patients, (iii) the temporal and spatial heterogeneity of genomic mutations that can be used for potential selection of matched therapies, (iv) the increasing use of immunotherapies where treatment assessment can be difficult by imaging (e.g., pseudo-progression), and lastly (v) technical advances in performing image-guided biopsies with increased accuracy and tissue quality. The need for the ever-increasing amounts of harvested tissues raises technical, logistical, and ethical challenges, most notably, (i) patient acceptance of repeat biopsies when decisions could be made with less invasive approaches, (ii) the accessibility of biopsy sites, (iii) the relatively high cost of sample allocation, distribution, and analyses often requiring different teams, and (iv) the long timeframe from tissue harvest to final data, often ranging from days to weeks. Therefore, what is needed are less invasive methods capable of analyzing cells rather than tissue cores. This in turn would be expected to lower complication rates and enable same day analysis as there would be no need for tissue embedding and sectioning. Together, such an approach could facilitate clinical workflows where treatment adjustments often cannot wait for weeks.

To address the above needs, we have been interested in developing, validating, and using analytical platforms to directly process cells in fine needle aspirates (FNA). FNA differ from core biopsies in that needles are much smaller (typically 21G as opposed to 17G), are less prone to causing complications and generally yield single cells or clusters of cells ready for point-of-care analyses. While cytopathology relies on the same sampling method, spectrally encoded chromogenic stains are limited in number and materials are often insufficient to process for both hematoxylin/eosin (HE) and immunocytopathology. Conversely, single cell analytical techniques^1–4^ are also feasible but are less commonly used in routine clinical practice given their relatively high cost, long turn-around times (weeks rather than hours to days), and current lack of reimbursement. Rather, these methods have become ones of choice for experimental studies.

We hypothesized that it should be possible to develop repeat single cell staining methods compatible with fresh samples on glass slides and within the same day of harvesting. We were particularly interested in imaging proteins since these are the primary drug targets, are generally more abundant compared to nucleic acids, can be analyzed within hours of sampling, and allow therapeutic efficacy assessment through phosphoprotein analysis. We initially tested several published methods^5,6^ but found that the relatively harsh conditions requiring oxidants for bleaching were not compatible with FNA-harvested cells. Optical bleaching methods for one to two channel imaging have been reported^7^ but we desired a more rapid multiplex readout for clinical applications. Alternatively, DNA barcoded antibodies have been used for chip-based analysis of scant cells^1^. However, we found that these methods had considerable background, were hard to quench with previously used photocleavable linkers^8^, and that short fluorophore-labeled DNA barcodes (e.g., 10–25 bp) showed problematic non-specific binding to nuclei when applied to cells for in situ hybridization and staining. We thus hypothesized that it should be possible to pre-hybridize fluorescent DNA imaging strands to matching mAb–DNA barcodes in vitro and use these reagents for cellular staining. Importantly, this approach provides a means for imaging-strand fluorochromes to be washed off and cells re-stained in subsequent cycles: because hybridization strength is dependent on salt concentration, optimized imaging strands can be stably attached to the barcoded antibody in PBS and rapidly cleared upon washing with deionized water. Here, we demonstrate that one such optimized method (SCANT; *s*ingle *c*ell *an*alysis for *t*umor phenotyping) is robust and can be used for pathway analysis in a clinical setting. Using PI3K⍺ isoform inhibitors in combination clinical trials^9–12^, we show dose-dependent target inhibition in different breast cancer models, stochastic heterogeneity, and clinical variability to response.

## Results

### Barcoding antibodies allows single cell imaging

**Fig. 1:**
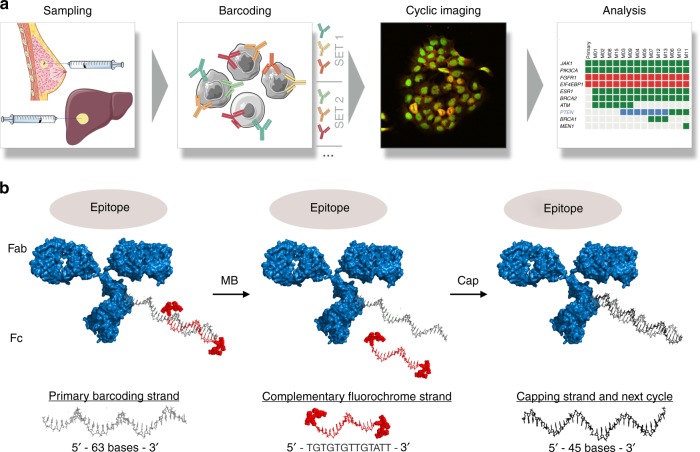
Overview of the method. **a** Cells obtained by image-guided fine needle aspiration are immersed in fixative, semi-permeabilized, and interrogated with antibody–DNA conjugates hybridized to complementary imaging strands that fluoresce in different channels. This cycle is repeated until all targets of interest are imaged. Automated image analysis allows graphing of data in analytical formats. **b** Barcoding approach. A 63 bp primary barcoding strand is attached to an antibody of interest using maleimide chemistry. In vitro, complementary imaging strands consisting of 13 bp and two fluorochromes are used to obtain fluorescent mAb–DNA conjugates. Cocktails of such antibodies with different fluorochromes (Pacific Blue, AF488, AF594, AF647) are then used to stain cells. After imaging, the fluorescent strands are simply washed off with melting buffer and the primary strands are capped to reduce additional cycle-to-cycle background

Figure 1 summarizes the approach for mAb–DNA barcoded FNA image analysis. Cells obtained by image-guided FNA are collected in PBS, briefly treated with collagenase, placed in a cytology fixative (10 min), transferred to the lab, and then processed for imaging or stored at −80 °C for later use. Cells are stained with mAb–DNA-Fl conjugates containing different fluorochromes for each mAb in each imaging cycle. These reagents are prepared prior to imaging per the design detailed in Fig. 1b, Supplementary Fig. 1 and Supplementary Table 1 and Supplementary Table 2. Briefly, an antibody of interest is: (i) functionalized with a short NHS-maleimide linker; (ii) reacted with a thiolated 63 bp primary barcoding strand; (iii) hybridized to a 13 bp doubly-fluorophore-labeled imaging strand to obtain the final mAb–DNA-Fl conjugate (Supplementary Table 2). Cocktails of such antibodies with complementary fluorochrome sets (e.g., Pacific Blue, AF488, AF594, and AF647) are then used to stain cells. After imaging, the fluorescent strands are simply washed with deionized water and the primary strands are capped with an unlabeled 45 bp blocking strand (Supplementary Table 2) to reduce background during subsequent rounds of imaging.

Prior to more extensive imaging, we performed a number of optimization, calibration, and validation experiments (Supplementary Fig. 2 and Supplementary Fig. 3). With the first iteration of mAb–DNA-Fl labeling conditions, we noted non-specific nuclear binding of imager strands, in spite of the antibody pre-hybridization approach (Supplementary Fig. 2, Supplementary Table 3). We attributed this to non-specific nuclear and mitochondrial DNA binding and DNA-imager strand attraction to positively charged histone proteins in the setting of a slow (but non-zero) off rate for the imager strand–barcode pair. We therefore performed additional experiments (Supplementary Table 3) to minimize non-specific signal. We varied DNA blocking conditions including the concentrations of Salmon Sperm DNA, poly-T blocker (24-mer), nonspecific blocker (random 24mer), buffer salts and Triton-X 100 at RT in a PBS-based protein buffer. These experiments resulted in an optimized protocol yielding high target-background ratios; this method (CSBx) was further shown to minimize background increase from cycle to cycle to a negligible level (Supplementary Fig. 4) and was thus used for all subsequent experiments. We next validated SCANT DNA-labeled antibodies by verifying that their localization matched the unmodified parent antibodies for a series of key targets (EGFR, S6, pS6, AKT, and pAKT) in A431 cells. Cells were stained with the native mAb followed by a fluorochrome-conjugated secondary antibody (green channel), washed, and then stained again with the SCANT mAb–DNA-Fl method (red channel), allowing for two-channel imaging to visually compare the simultaneous staining and enable statistical analysis. These results show excellent co-localization as seen in Supplementary Fig. 3 (mean Pearson’s *R* value 0.94). Additional experiments were performed to exclude the possibility of artifactual colocalization from the primary/secondary antibody staining process (Supplementary Fig. 3B). Next, we compared target quantification via SCANT in cell-line derived cohorts of single cells to flow cytometry (*R*^2^ = 0.85) and ELISA across a selection of antibody/cell line targets (*R*^2^ = 0.88; Supplementary Fig. 5). This data showed good correlation between overall protein levels measured by gold standard methods as compared to the SCANT imaging method.

**Fig. 2:**
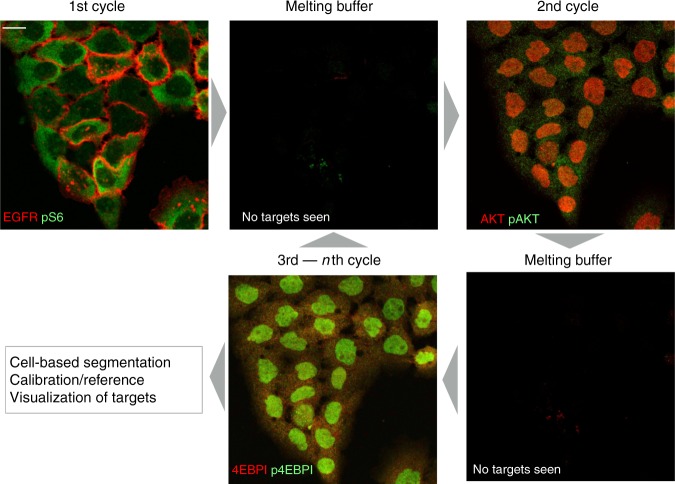
Serial imaging of multiple targets within the same cell. mAb–DNA conjugates against EGFR and pS6 were used for the first round of staining in cultured A431 cells (pseudocolored with red and green, respectively). Note the expected location of each protein. Within minutes of addition of the melting buffer (deionized water), the targets are no longer detectable by imaging. In the second cycle, mAb–DNA conjugates against AKT and pAKT were imaged (again, pseudocolored with red and green). The second round of melting buffer was used to dissociate the imaging strands. During the third cycle of imaging, 4EBPI (red) and p4EBPI (green) were imaged in the same cells. The cycles can then be repeated until all desired markers have been analyzed. Scale bar (top left) represents 20 µm

**Fig. 3:**
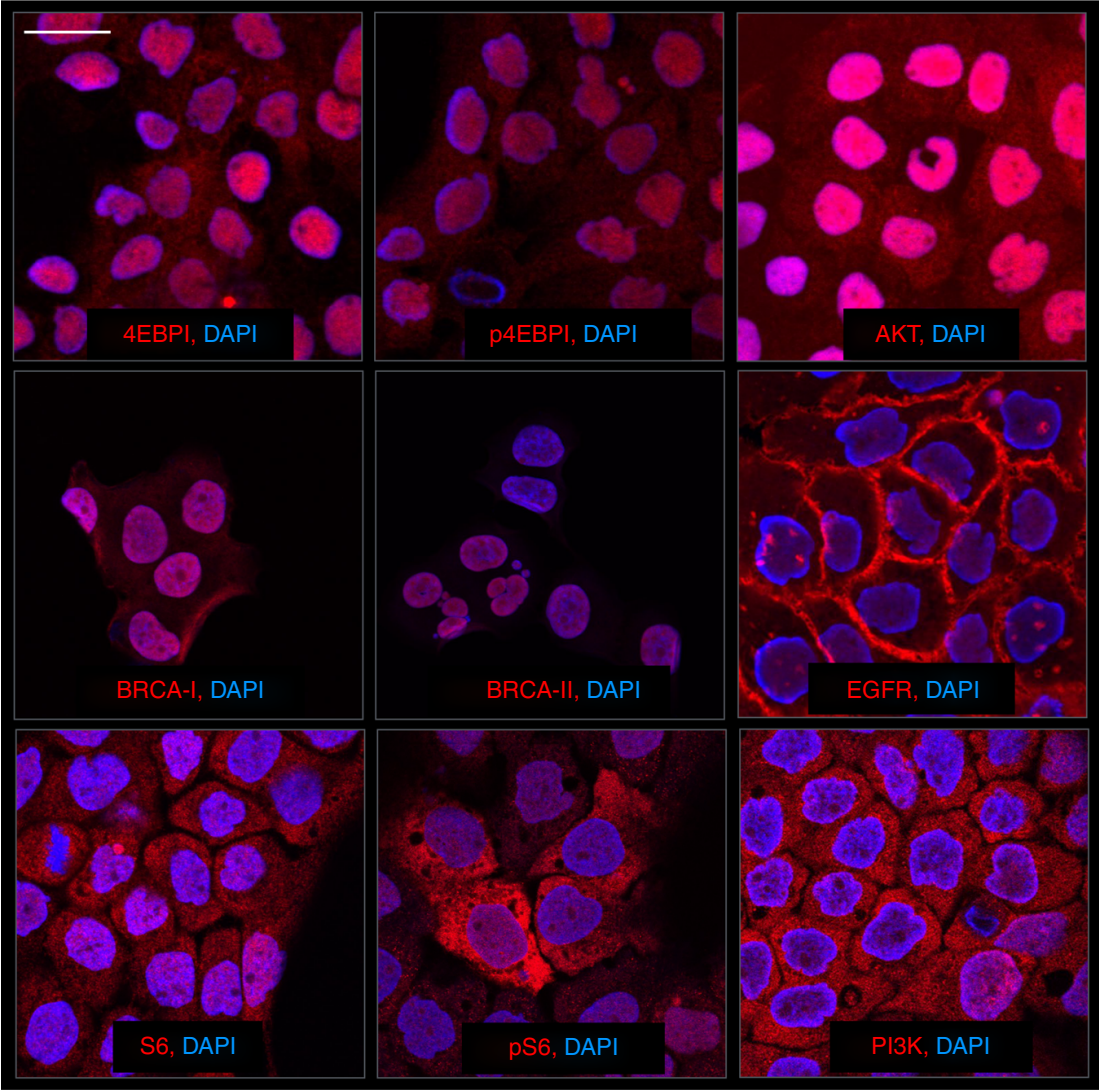
Staining of different cellular targets of interest. Representative examples of cultured A431 cells stained with DAPI to highlight nuclei. Different mAb–DNA conjugates were used to reveal primary targets shown. Scale bar (top left) represents 30 µm

Figure 2 shows one representative example of target staining in cultured cells. We focused on relevant targets in the PI3K pathway and conducted preliminary cycling experiments where we stained cells with two color cycles targeting, (i) EGFR and pS6 (note cell membrane and cell cytosol targeting), (ii) AKT and pAKT, and (iii) 4EBPI and p4EBPI. During all cycles, we noted negligible nuclear background artifact and excellent washout of fluorochromes for each respective target. Additional examples of target staining from the broader mAb–DNA-Fl panel are shown in Fig. 3 and Supplementary Fig. 6.

### SCANT allows detailed analyses of phosphorylation

**Fig. 4:**
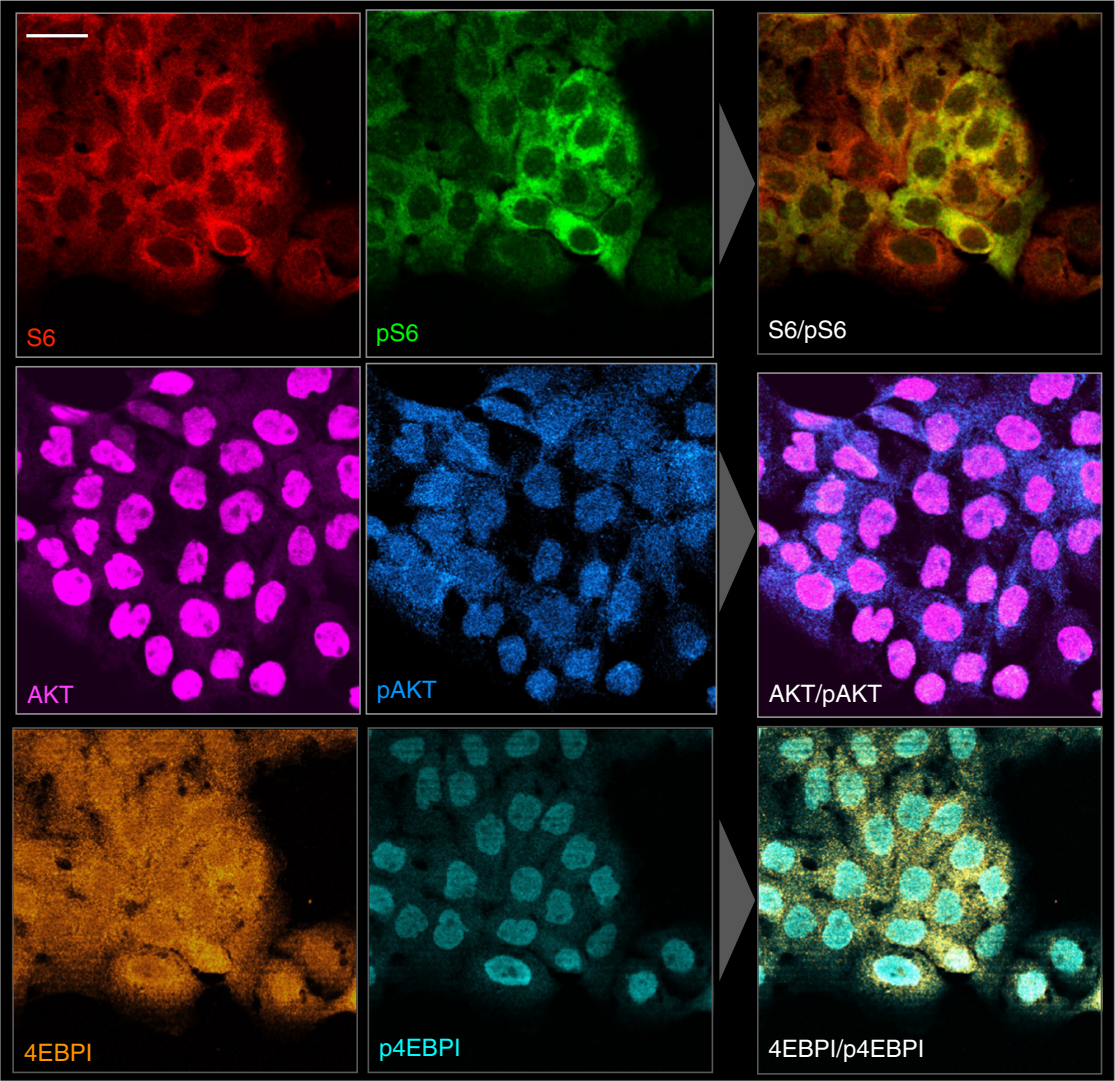
Phosphoprotein analysis in single cells. Representative examples of phosphoprotein ratio imaging for S6/pS6RP (calculated corrected ratio of pS6/S6 of 0.41), AKT/pAKT (calculated ratio of pAKT/AKT of 0.16), and 4EBP1/p4EBP1 (calculated ratio of p4EBP1/4EBPI of 0.3) in cultured A431 cells. Scale bar (top left) represents 50 µm

We next set out to analyze the phosphorylation status of different targets of interest: S6, 4EBP1, and AKT. Figure 4 summarizes one representative example in cultured cells. All proteins of interest were identified in their expected subcellular location. For example, 4EBP1 was located in the nucleus and cytoplasm whereas the phosphorylated 4EBP1 was primarily located in the nucleus. Reciprocal morphologic differences were observed with AKT/pAKT, with the phosphoprotein preferentially localized to the cytoplasm (Fig. 4).

To understand the dynamic range of our antibody phosphorylation targeted DNA-conjugates, we conducted additional experiments in MCF-10A mammary epithelial cells to quantify S6 phosphorylation as a function of growth factor stimulation (Supplementary Fig. 7). Serum starvation was followed by titrated EGF treatment, fixation, and mAb–DNA-Fl imaging of pS6 levels. These experiments illustrated the measurement of pS6 at 0 nM EGF, with increasing phosphorylation levels as EGF concentrations approached those in normal MCF-10A growth media (20 nM). From this work, we concluded that our DNA-conjugate–antibodies were indeed able to measure both low and high phosphorylation levels.

While visual inspection of images has been the mainstay of cytopathology, we also wanted to automate image analysis to display and analyze data across cohorts of cells and in clinical settings in semi-automated fashion (Supplementary Fig. 8 and Supplementary Fig. 9). We developed an artificial intelligence algorithm that automatically segments objects and categorizes them via a convolutional neural network (CNN) into either tumor cells of interest or host cells (the latter based on molecular host cell markers, see below)^13–16^. Due to the large variety of sample qualities obtained with FNAs, we designed the algorithm with a maximum amount of flexibility via the creation of a training set of tumor cells from 9 heterogeneous patient samples with ~800–2000 cells from each.

### Pathway analysis in cancer cells

**Fig. 5:**
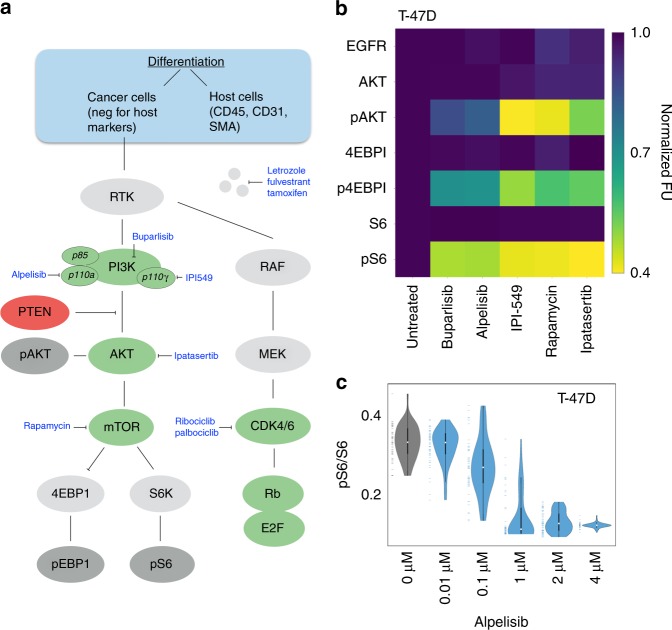
Pathway analysis and therapeutic intervention. **a** Clinical sample analysis. Harvested cells are first differentiated into host and cancer cells and subsequent analyses are performed on the latter. Primary drug targets are shown in green. **b** Image analysis of ~500 T47D cells before/after exposure to targeted therapeutics showing initial response normalized to 1 and their measured, corrected fluorescence response according to the described scale bar. **c** Dose–response analysis of alpelisib (BYL-719), a selective PI3K inhibitor of p110α. The violin plots show sample heterogeneity to response and are consistent with an IC50 of <1 μM in the T47D cell line

Using the automated image analysis SCANT protocol, we next set out to perform more extensive analyses across different cell lines and drug targets in the PI3K pathway. We tested a number of different targeted drugs (PI3K: alpelisib (BYL719), buparlisib (BKM120) and IPI549; AKT: Ipatasertib (GDC-0068); mTOR: rapamycin) for their effects on T47D breast cancer cells (Fig. 5). As can be seen, all drugs had profound effects on the phosphorylation status of AKT, 4EBP1, and S6. Figure 5c summarizes one representative example of a dose response experiment using SCANT analysis. For each dose tested, we imaged ~100 cells, processed them through the above-described algorithm and plotted the data as shown. The violin plots clearly demonstrate the heterogeneity of protein expression and dose-dependent suppression of S6 phosphorylation. From this data, an IC_50_ of alpelisib (BYL-719) was estimated to be ~100 nM, consistent with literature reports^17^. Additional experiments in other breast cancer cell lines (HCC1937, HCC1954) are shown in Supplementary Fig. 10. The comparative alpelisib potency data shows differences across the three cell lines tested with the most pronounced effects in T47D cells, again consistent with literature reports^17–19^.

### Separating host and cancer cells in clinical specimens

**Fig. 6:**
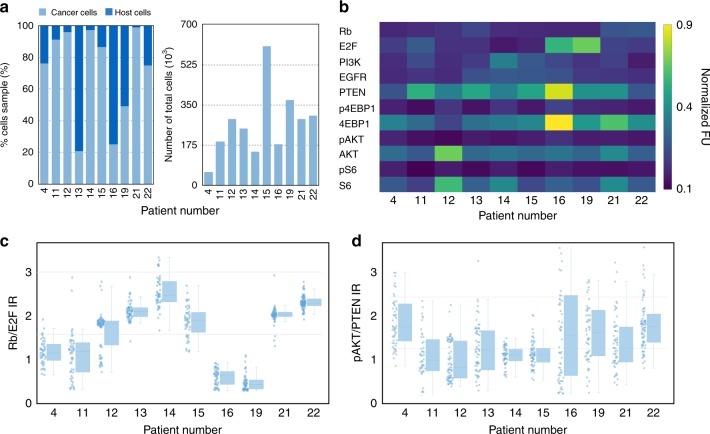
Analysis of clinical samples. **a** Calibration experiments to identify the composition of samples. Host cell analysis of clinical samples demonstrates that tumor cells made up a large fraction of the cells within FNAs. However, there was considerable heterogeneity amongst patients. **b** Heterogeneity of key drug targets measured in pretreatment clinical samples. **c** Analysis of the ratio of Rb/E2F shows the heterogeneity within a given patient and also between patients. **d** Measurement of pAKT/PTEN ratios in the same patients displays increased cell-to-cell variability but less overall heterogeneity

The above optimization and characterization experiments were all performed in well-characterized cancer cell lines grown at known density and exposed to known concentrations of single drug treatments to unequivocally validate single cell imaging observations. Clinical specimens differ in that samples contain not only cancer but also host cells and in that cells may be sparsely distributed. To separate host and cancer cells, we stained all cells with a cocktail of antibodies for CD45 (leukocytes), CD31 (endothelial cells), and SMA (fibroblasts). We assumed that all cells negative for this cocktail were likely primary cancer cells and verified the performance of the SCANT method by flow cytometry and immunofluorescence imaging. Finally, we analyzed 10 clinical samples with notably high post-processing cell counts and found a host/tumor cell ratio ranging from ~95% tumor cell/5% host cell ratio to ~ 25% tumor cell/75% host cell ratio (Fig. 6a).

In traditional cytopathology, FNA smears are often obtained and cells are analyzed following HE staining. While we attempted this approach with SCANT, we found that the image quality was generally substandard, that cells were distributed over a too large field of view (FOV) and that cell clusters impeded the automated image analysis approach. We therefore obtained single cell solutions following mild collagenase treatment (to dissociate any cell clumps) prior to fixation and imaged cells in a defined, concentrated FOV.

### Biomarker expression in clinical samples

To analyze scant clinical FNA samples, we asked a number of questions: (i) what is the protein expression profile of biomarkers of interest in clinical samples; (ii) how divergent are protein profiles within a cancer (intra-tumor); and (iii) how divergent are protein profiles amongst patients (inter-patient)? Figure 6b summarizes the expression levels of key proteins in the PI3K pathway for 10 pre-PI3K therapy patients.

Since our ultimate goal was to image drug effects, we also performed in-depth analyses on key downstream markers. Figure 6c,d summarizes expression ratios of Rb/E2F and pAKT/PTEN as indicators of key PI3K- and CDK4/6-relevant proteins. As can be seen from the data, there were higher levels of heterogeneity in Rb/E2F ratios as compared to pAKT/PTEN ratios from patient to patient. Of note, when comparing phosphorylation and total protein levels between cells derived from FNAs and those derived from cell culture, we observed similar magnitudes of fluorescence intensity across a number of proteins (Supplementary Fig. 11).

### Analysis of treatment response

**Fig. 7:**
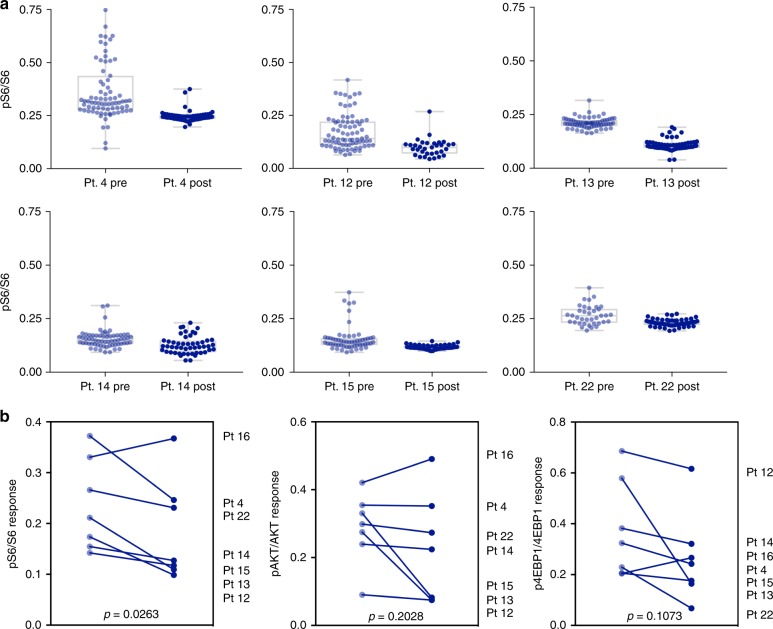
Patient treatment response. **a** Single cell analysis of 6 responder patients showing pS6/S6 ratios at baseline (off therapy) and following clinical trial dosing. Pre- and post-treatment single cell values were compared by Mann–Whitney *U* test (*P* < 0.001 for all six samples). **b** Summarized responses for all analyzed patients before and after treatment for pS6/S6, pAKT/AKT, and p4EBP1/4EBP1 ratios. *P*-values for the patient sample means were calculated with a ratio paired, 2-tailed *T*-test

Having performed the above experiments we next set out to serially analyze FNA samples from 7 patients before and after enrollment into a clinical trial (Fig. 7, Supplementary Fig. 12). This trial (NCT01872260) involved a study of triple therapy with BYL719 (PI3K-⍺ inhibitor), LEE011 (CDK4/6 inhibitor), and letrozole in advanced ER^+^ breast cancer. As a generic readout of pathway inhibition, we determined pS6/S6 ratios in pre- and post-treatment samples. Our data show (Fig. 7) that ratios and heterogeneity generally decreased in 6 patients (−18% for Pt 4, −25% for Pt 12, −50% for Pt 13, −10% for Pt 14, −5% for Pt 15, −15% for Pt 22). In one additional patient, there was an increase (30% for Pt 16) in the pS6/S6 ratio (Supplementary Fig. 12). Differences in this patient, including FNA site (T2 vertebra versus soft tissue), PI3K mutation status (wildtype) or overall patient responsiveness to therapy may have played a role in this patient’s phosphorylation dynamics.

## Discussion

The current study was designed to rapidly obtain single cell information on key proteins or pathways in clinical specimens obtained by FNAs. With this method, it is possible to harvest 10^2^–10^4^ individual cells per pass depending on technique and tissue type. During a typical image-guided procedure, 2–4 passes are obtained effectively yielding thousands of cells. The cellular analysis is imaging-based and results in multiplexed information on as many cells as typical microscopes are capable of visualizing.

We argued that image-guided tumor biopsies are generally well tolerated, have low complication rates, and can therefore be performed repeatedly^20^. Cytopathology of similarly obtained specimens is used routinely in the clinic but often limited to HE stains to determine the presence or absence of malignancy. Multiplexed imaging of cells adhered to glass slides has been much more difficult because of the harsher methods required for fluorescence bleaching or fluorochrome release^5^. In search of more gentle methods, we decided to utilize DNA bar-coded antibodies where the hybridized imaging strand could be easily washed off samples with deionized water. Here, we show that this method works extraordinarily well and allows for comprehensive profiling of harvested cells at low costs and complexity.

With all harvested samples, the first question is always one of origin. All of our samples were obtained under image guidance where needle placement was continuously monitored during aspiration. This assured that the majority of cells were obtained from within the tumor rather than the surrounding parenchyma. The second question generally is whether tumoral cells are of cancer or host cell origin. While we initially entertained the use of positive (e.g., quad marker^21^) and negative (e.g., CD45+) selection methods using magnetic beads, we realized that these methods resulted in considerable loss of scant cells. We therefore developed an unbiased imaging approach where we imaged all cells and defined host cells by CD45 (lymphoid and myeloid cells), CD31 (endothelial origin), and SMA (fibroblast) positivity. This imaging approach allows spatial registration of host and tumor cells on the same slide.

The above approach allowed us to preferentially analyze pathways in tumor cells. We used a new combination of an artificial intelligence approach and multiplexed imaging to segment and classify cells automatically, so that results could be displayed in graph or pathway format rather than through visual inspection. We applied this method to profiling of metastatic breast cancer as well as assessing treatment response to PI3K inhibitors. PIK3CA, which encodes the p110⍺ catalytic subunit of PI3K, is strongly implicated in oncogenic PI3K signaling^22^ and the considerable frequency of PIK3CA mutations suggests a therapeutic role for PI3K⍺ inhibitors^23,24^. Several pan-PI3K pathway inhibitors are in development but more selective PI3K⍺ inhibitors are of interest to minimize side effects, provide a wider therapeutic window, and allow for combinations with other therapies such as the CDK4/6 inhibitor and letrozole as tested here^9,10,25^. Our clinical feasibility data show, (i) using the SCANT method, it is possible to measure multiplexed protein analyses in clinically variable patient samples; (ii) in the majority of patients analyzed, PI3K markers and ratios such as pS6/S6, pAKT/AKT, and p4EBP1/4EBP1 showed a response to drug dosing as measured by FNA and the SCANT method; and (iii) there is considerable heterogeneity between patients in terms of the relative levels of individual proteins and phospho-proteins, as well as in the ratios of metrics which could be considered tumorigenic such as Rb/E2F. The observation of a statistically significant therapy-associated response in the pS6/S6 ratio (*p *= 0.026) represents a potentially promising starting point for a pathological clinical read out in future work, as it leverages ratiometric measurement of paired biopsy samples to correct for cell-to-cell and patient-to-patient variability in absolute signal intensity. Reference cell populations with defined phosphorylation states (e.g., unstimulated MCF-10A (low) and T47D (high)) would provide facile negative and positive controls for assay validation at the time of pathology interpretation.

Going forward, it should be easy to expand the palette of antibodies for analysis of other proteins and therapeutic targets/pathways. For example, an obvious application is the analysis of immune cells within tumors. To this end, it should be possible to derive T-cell, B-cell, and myeloid cell profiles from FNA. Such an approach is aided by the recent development of single cell analytics and validation of cell markers of new immune cell subtypes^26–28^.

## Methods

### Study design

The objective of this research was to develop a multiplexed platform for imaging cellular biomarkers of interest in clinical samples and in cell lines with a focus on understanding treatment response in cancer. We hypothesized that protein networks (as opposed to single biomarkers) will reveal interesting insights into how cancers evolve and respond to drugs. All in vitro studies were performed in replicates (typically *n* = 3 unless otherwise specified). Following optimization, studies with the final protocol were repeated multiple times on different days to ensure reproducibility.

Following extensive cell line validation, clinical feasibility studies were performed on a limited cohort of patients. We selected the number of patients based on a 1-year enrollment cycle. All experiments on clinical studies were performed blinded during experimental procedures and raw data analysis.

### Antibodies

Antibodies were purchased from commercial sources (Cell Signaling Technology, Abcam, Novus Biologicals, Bristol Myers Squibb) as summarized in Supplementary Fig. 1. Prior to DNA conjugation, all antibodies were tested on cell lines in typical immunocytochemistry experiments (with a fluorescent secondary antibody) to understand the binding and localization of each unmodified antibody with respect to its target. DNA conjugates were directly compared to immunocytochemistry results to ensure correct localization for each antibody.

### DNA sequence design

Custom designed oligos were purchased from Integrated DNA Technologies. Supplementary Table 2 summarizes the different oligos used for barcoding, imaging, and capping as detailed in Fig. 1. In brief, the primary conjugation strand was 63 bases, the imaging strand 13 bases, and the capping strand was 45 bases. The imaging strand sequence was designed to hybridize stably (*T*_m_ > 37 C) in PBS ([Na^+^] = 140 mM) and dissociate rapidly (*T*_m_ < 20 C) under low salt conditions (<5 mM Na^+^), allowing facile image cycling at room temperature. The length of the capping strand was selected for tight binding under either PBS/low salt conditions so that it remains hybridized during subsequent image cycles. Secondly, sequences were designed to minimize homology with the human genome (NCIBlast). In order to simplify antibody/DNA sets, we focused on one single optimized generic DNA sequence, although we expect that a variety of DNA sequence designs would work in a similar manner.

### DNA–antibody conjugation

Antibodies (Supplementary Table 1) were obtained in PBS (with or without sodium azide). Antibodies were concentrated to 1–3 mg/mL using Amicon (100 K MWCO) Ultra centrifugal filters. All antibodies were then solvent exchanged with Zeba spin desalting columns (7 K MWCO) into a PBS-bicarbonate buffer (pH 8.4) to remove azide and establish the optimal pH for efficient amine labeling. Antibodies were then incubated at pH 8.4 with 20–60 equivalents of MAL-dPEG-NHS ester (Quanta Biodesign, product #10266) in 10% DMSO at RT for 20 min. Afterwards, excess reagents were removed using a Zeba spin desalting column (7 K MWCO, PBS). Thiol-modified 63-bp DNA barcodes were reduced using tris(2-carboxyethyl)phosphine (TCEP, 25 molar excess over DNA) in PBS for 2 h at RT. The reduced DNA oligos were then purified using Zeba spin desalting columns (7 K MWCO), with PBS as a wash buffer.

In a typical conjugation process, a 20-fold molar excess of DNA oligos were incubated with maleimide-activated antibodies. The conjugation reaction was allowed to proceed for 12 h at 4 °C. DNA barcode–antibody conjugates were purified using a 100 K MWCO Amicon Ultra centrifugal filter followed by 3 washes with PBS.

UV/vis absorption spectra of DNA barcode–antibody conjugates were then measured via Nanodrop; the degree of labeling (DOL) was calculated from the measured A260/A280 ratio. Briefly, UV absorption spectra of the DNA barcode and antibody were measured and used to calculate full pairwise values for the extinction coefficient of each species at both 260 and 280 nm. We then derived an explicit solution for the absorbance ratio (260/280 nm) as a function of stoichiometric composition—in the absence of any DNA this matches the ratio of the parent antibody, and as the DNA labeling ratio increases, this approaches the values of the parent DNA.

$${\mathrm{DOL}} = \frac{{\varepsilon 260_{{\mathrm{Ab}}} - {{R}} \ast \varepsilon 280_{{\mathrm{Ab}}}}}{{{{R}} \ast \varepsilon 280_{{\mathrm{DNA}}} - \varepsilon 260_{{\mathrm{DNA}}}}}$$

where $$R = \frac{{A260}}{{A280}}$$

### DNA–fluorochrome conjugation and mAb–DNA hybridization

Imaging strand oligonucleotides (13 bp) doubly-labeled with green, red, and far-red fluorochromes (AlexaFluor 488 (AF488), AlexaFluor 594 (AF594), and AlexaFluor 647 (AF647)) were purchased from IDT. Pacific Blue oligos were prepared from the corresponding bis-amino DNA (10 nmol) and PacBlue-NHS (75 equivalents, from a 50 mM stock solution) in borate buffer (20 mM, pH 8.5) supplemented with DMSO (to achieve a final composition of 50% DMSO) and 25 mM NaHCO_3_. PacBlue-labeled imaging strands were purified with Glen Gel-Pak columns.

Fluorophore-labeled imaging strands, each complementary to the MAb–DNA barcode, were incubated at a 1:1 ratio for 20 min in PBS at RT. To purify MAb–DNA-Fl conjugates, the reaction mixture was subjected to 3× Zeba column filtering (40 K MWCO) and eluted in PBS for storage. Purified mAb–DNA-Fl conjugates were measured via nanodrop to verify the expected DOL.

### Cell lines

Validation and drug analysis studies for SCANT were performed in A431, HCC-1937, HCC-1954, MCF-10A, and T47D cell lines. All cell lines were purchased from the American Tissue Culture Collection (ATCC). Cells were passaged in medium prepared to the specifications of each individual cell line according to ATCC. Cell lines were routinely tested for mycoplasma contamination using the PlasmoTest Mycoplasma Detection Kit (InvivoGen).

### Drug treatments of cell lines

To test the effect of drug treatment on protein and phosphoprotein levels, cell lines were dosed with drugs focused on the PI3K pathway including buparlisib (BKM-120, Selleck Chemicals), alpelisib (BYL-719, Selleck chemicals), IPI-549 (Selleck Chemicals), sirolimus (rapamycin, Selleck Chemicals), and ipatasertib (GDC-0068, Selleck Chemicals). To determine dosing for imaging-based experiments, all drugs were screened against each cell line to determine IC_50_ values via conventional cell viability assay (MTT). Treatments were administered at 80% of the IC_50_ dosing overnight prior to fixation for cell line treatments (Fig. 5b, Supplementary Fig. 10).

### Clinical samples

The study was approved by the Institutional Review Board at the Dana Farber/Harvard Cancer Center (HCC13-367, HCC13-416) and informed consent was obtained from all subjects. Seventeen minimally invasive FNAs were obtained in the 10 patients as part of a routine workup including coaxial core biopsies and additional FNA for cytopathology. All patients had advanced stage, metastatic ER+ breast cancer. All pre-treatment biopsies were collected during a treatment hiatus. All post-treatment biopsies were collected during the first cycle of BYL719/LEE001/letrozole treatment (Novartis; NCT01872260).

The FNA were obtained coaxially with 21G needles and prior to the routine core biopsies. Correct needle location was confirmed by ultrasound or CT imaging. FNA samples were briefly subjected to collagenase to break apart samples and then added to a Lyse/Fix buffer (BD Biosciences) for 10 min at 37 °C and washed twice with PBS with 2% BSA. All subsequent centrifugations were performed at 300*g* for 5 min. A total of 17 samples were prepared and analyzed independently via the SCANT method. In addition, aliquoted clinical samples were stored at −80 °C.

### Sample preparation

Cells from culture or FNA were added to slides after processing with a Cytospin system (Thermo Scientific). Cells were permeabilized and blocked in a solution of Odyssey blocking buffer (LI-COR Biosciences), 1 mg/mL Salmon Sperm DNA (Sigma Aldrich, D7656) and 25 µM poly-T blocker (24-mer), and 0.1% Triton-X 100 at RT. All fixed cells were then subjected to primary mAb–DNA-Fl labeling followed by cycling. The workflow for samples included ~30 min for collagenase treatment of samples and fixation in a lyse fix buffer, followed by cytospinning samples onto a slide (~5 min). Samples were then prepared for cycling via a 2 h pre-blocking step. Each detection cycle was carried out over a 45 min antibody incubation followed by a 30 min period for fluorophore washing and DNA strand capping. Total sample preparation times varied based on the number of proteins analyzed, but generally ranged from 4 to 8 h. The hands-on time was substantially shorter (~1 h). Irrespective of the protocol, the analysis allowed same day turn-arounds for faster therapeutic decision making, something sorely missed in current clinical practice.

### Fluorescent imaging

Images were acquired on either an Olympus BX-63 upright automated epifluorescence microscope, used for high throughput imaging, or an Olympus FV1000 confocal laser microscope used for high magnification imaging. On the BX-63, Pacific Blue, AF488, AF594, and AF647 probes were excited via traditional DAPI, FITC, TRITC, and Cy5 filter cubes, respectively, while on the FV1000 these probes were excited by 405, 473, 559, or 620 nm lasers in combination with appropriate beam splitters (SDM473, SDM560, and/or SDM 640) and emission filters BA430–455, BA490–540, BA575–620, BA575–675, and/or BA655–755 (Olympus). To create aligned images of the same local areas across cycles, landmarks on slides were utilized for initial image alignment followed by final image registration in ImageJ.

### ELISA

NuncMaxiSorp plates (44-2404-21; ThermoFisher Scientific) were coated overnight at 4 C with 5 µg of cell lysates. Plates were then washed with PBST (0.005% Tween-20) and blocked with 1% BSA for 1 h at room temperature. Following blocking, plates were washed again with PBST and then incubated with appropriate dilutions of primary antibodies in 1% BSA for 2 h at room temperature. After this step, plates were washed again with PBST and incubated with HRP-labeled secondary antibodies (1:500 dilution) in 1% BSA for an hour. Finally, plates were washed with PBST and incubated with 1 Step Ultra TMB ELISA substrate (34028B; ThermoFisher Scientific) and reaction was quenched with 0.1 M HCl once the color development had saturated (max incubation time was 30 min). The absorbance was measured at 450 nm using Tecan plate reader.

### Flow cytometry

Cell lines were fixed with 4% paraformaldehyde for 15 min at room temperature, washed with PBS, and then permeabilized on ice for 30 min with 90% methanol. Cells were then washed with PBS supplemented with 0.5% BSA. 500,000 cells/marker were incubated with primary antibodies (at vendor suggested dilutions) for 30 min at room temperature. Cells were then washed and incubated with AlexaFluor 488® conjugated secondary antibodies (2 μg/mL) for 30 min at room temperature and then washed again. Fluorescent signals were measured using BD LSRII Flow Cytometer (BD Biosciences). Mean fluorescent intensities (MFIs) were normalized using the formula (signal primary antibody − signal IgG isotype control)/signal secondary antibody. Each washing step comprised of three 5 min washes at 300*g* with PBS supplemented with 0.5% BSA.

### Separating tumor cells from host cells

FNA samples of human tumors invariably contain host and tumor cells. In order to limit pathway analysis to cancer cells, we developed a method to first identify host cells. This was done by staining the entire cell population with a cocktail of antibodies all containing the same fluorochrome (AF405): anti-CD45 (leukocytes), anti-CD31 (endothelial cells), anti-SMA (fibroblasts); Supplementary Table 1. In preliminary feasibility experiments, we determined that this imaging-based method proved reliable and did not result in cell loss as compared to negative and positive cell selection methods using magnetic beads.

### CNN development

Classification of individual cells was completed via the development of a CNN-based algorithm^15^. Briefly, we compiled a set of co-registered cycled images of patient FNA samples. Co-registration was completed via the FIJI/ImageJ algorithm StackReg.

To create a training set for the CNN, individual cells from multiple patients were identified from coregistered images utilizing an in-house developed watershed and segmentation algorithm based on previous work^29,30^. Individual cells were manually classified as either host cells (44% of the total) or tumor cells (56% of the total) based on human interpretation of the presence or absence of an exclusion stain composed of CD31/CD45/SMA.

To create a classifier, we utilized the previously established VGG16 architecture (Supplementary Fig. S9A)^16^, created in Python (Keras with a Tensorflow backend). Analysis and verification of the data set was completed via a train/test/validate split of 80/10/10 among a total sample size of ~10,000, where we found an accuracy of 92%. For the accuracy measures of the network, the 10% of samples utilized were unseen by the algorithm until evaluation. Furthermore, in testing this system against prepared mixtures of cultured Daudi cells and Caco2 tumor cells of varying ratios, we found a high degree of accuracy between algorithm interpretation of stained samples versus the counted ratios initially placed in the mixtures (Supplementary Fig. 9B).

### Patient sample classification and quantitative analysis

To analyze patient samples, cycled images were first co-registered, and individual cells were identified, as above. Cells were then classified via the described CNN algorithm into either tumor cells or host cells. Following cell identification and classification, a rolling ball background subtraction algorithm was applied to each image for downstream analysis.

For identified tumor cells, individual images composing each marker/cycle were then separated into individual images. For each image, a local adaptive thresholding algorithm was applied to determine the unique staining pattern for that individual marker in a specific cell, and a raw intensity value was obtained for the identified region of interest.

Raw image intensities were processed through a multi-step normalization procedure to account for the individual DOL and staining efficiency of each labeled mAb–DNA-Fl. Specifically, intensities were scaled to a correction factor derived for each primary antibody in standardized cell line imaging experiments. Under matched conditions, we compared the brightness of on-target staining yielded by the mAb–DNA-Fl to that of the parent antibody recognized by a fluorescently labeled secondary antibody standard. By directly comparing the in situ brightness, this master correction ratio accounts for not only DOL, but also variation in fluorophore brightness, microscope filter cubes, and any impact of the DNA labeling chemistry on the antibody binding performance. Measurements were further normalized for their respective fluorophore according to a calibration function of fluorescent intensity versus exposure time (Supplementary Fig. 5B).

### Statistical analysis

*P*-values for patient sample means were calculated by first conducting a ratio paired, 2-tailed *T*-test for each phospho/total marker pair (S6, AKT, 4EBP1) in GraphPad Prism. Secondly, within each patient’s cell population we utilized a Mann–Whitney test to test for significance between pre- and post-treatment single cell values, also in GraphPad Prism. Pearson *R* values for images were calculated using the Coloc2 module in FIJI.

### Code availability

All code used in this project is publicly posted in GitHub under https://github.com/rjgiedt/SCANT. Code was tested in Python version 3.6. Detailed instructions are available in the code repository.

## Electronic supplementary material

Supplementary Information

## Data Availability

The datasets generated during and/or analyzed during this study are included in this published article (and its supplementary information files) and/or are available from the corresponding author on reasonable request.
