# Supplementary material for: Single-cell barcode analysis provides a rapid readout of cellular signaling pathways in clinical specimens
Source: Nat Commun. 2018 Oct 31;9:4550. doi: 10.1038/s41467-018-07002-6 (PMC6208406; doi:10.1038/s41467-018-07002-6)
Supplement: Supplementary file 1 — Supplementary Information [file 41467_2018_7002_MOESM1_ESM.pdf]

Supplementary Information

Single-cell barcode analysis (SCANT) provides a rapid readout of cellular signaling pathways in clinical specimens

Randy J. Giedt[1†], Divya Pathania[1†], Jonathan Carlson[1,3†], Phil McFarland[1], Andres Fernandez del Castillo[1], Dejan Juric[3], Ralph Weissleder[1,2,*]

[1] Center for Systems Biology, Massachusetts General Hospital, 185 Cambridge St, CPZN 5206, Boston, MA 02114,
[2] Department of Systems Biology, Harvard Medical School, 200 Longwood Ave, Boston, MA 02115
[3] MGH Cancer Center, Massachusetts General Hospital, 185 Cambridge St, CPZN 5206, Boston, MA 02114,

[†] These authors contributed equally to the manuscript

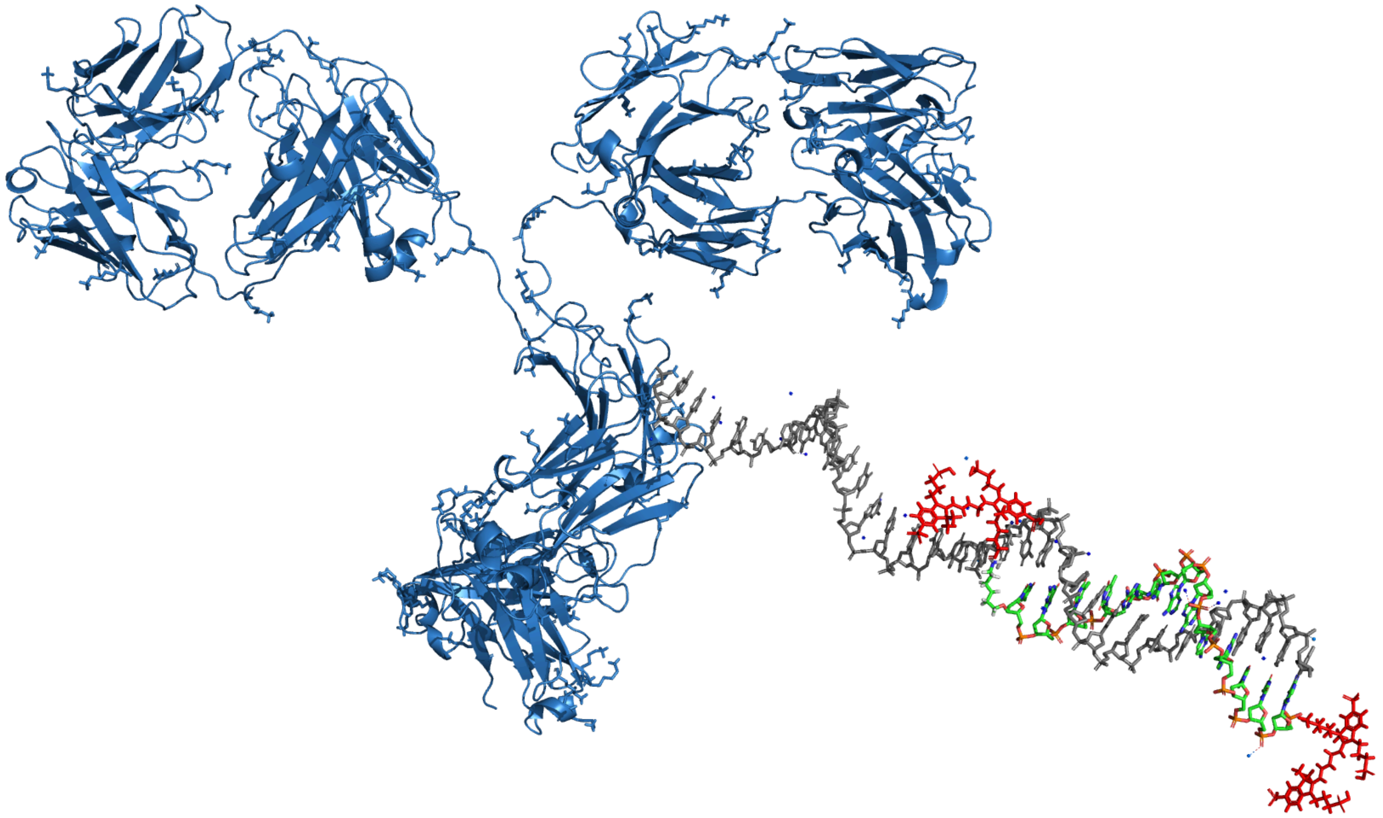

Supplementary Figure 1: Barcode structure. Structure of a representative DNA-barcoded antibody and hybridized fluorescent imaging strand, with AF647 tags in red. For compactness only 32 of the 63 basepairs of the barcode (gray) are shown. (PDB IDs 1IGT, 3BSE)

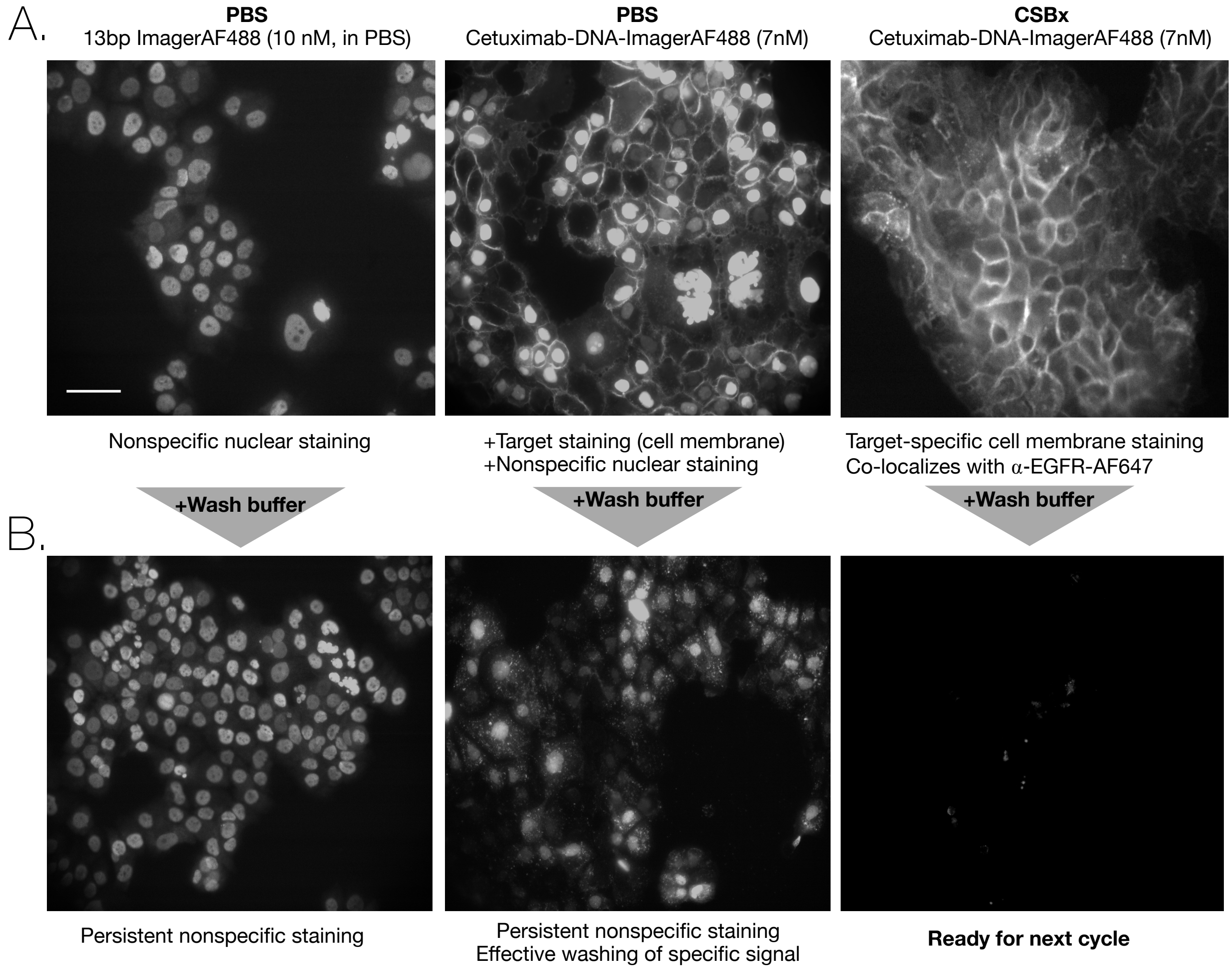

Supplementary Figure 2: Comparison of buffers for staining (A) and de-staining conditions (B). A. The 13bp imaging strand alone (left panel) or the mAb-DNA-AF488 resulted in high nuclear off-target staining as shown here for EGFR staining in cultured A431 cells (middle panel). However, with the use of the optimized CSBx buffer (Table S3), only target staining is observed (right panel; see also Figure S3 for further validation). **B**. Washout of accumulated non-specific staining was difficult under water rinsing conditions, but had excellent performance following optimized sample blocking, yielding efficient image cycling and high fidelity staining of specific targets. Scale bar (top left) represents 75 μm.

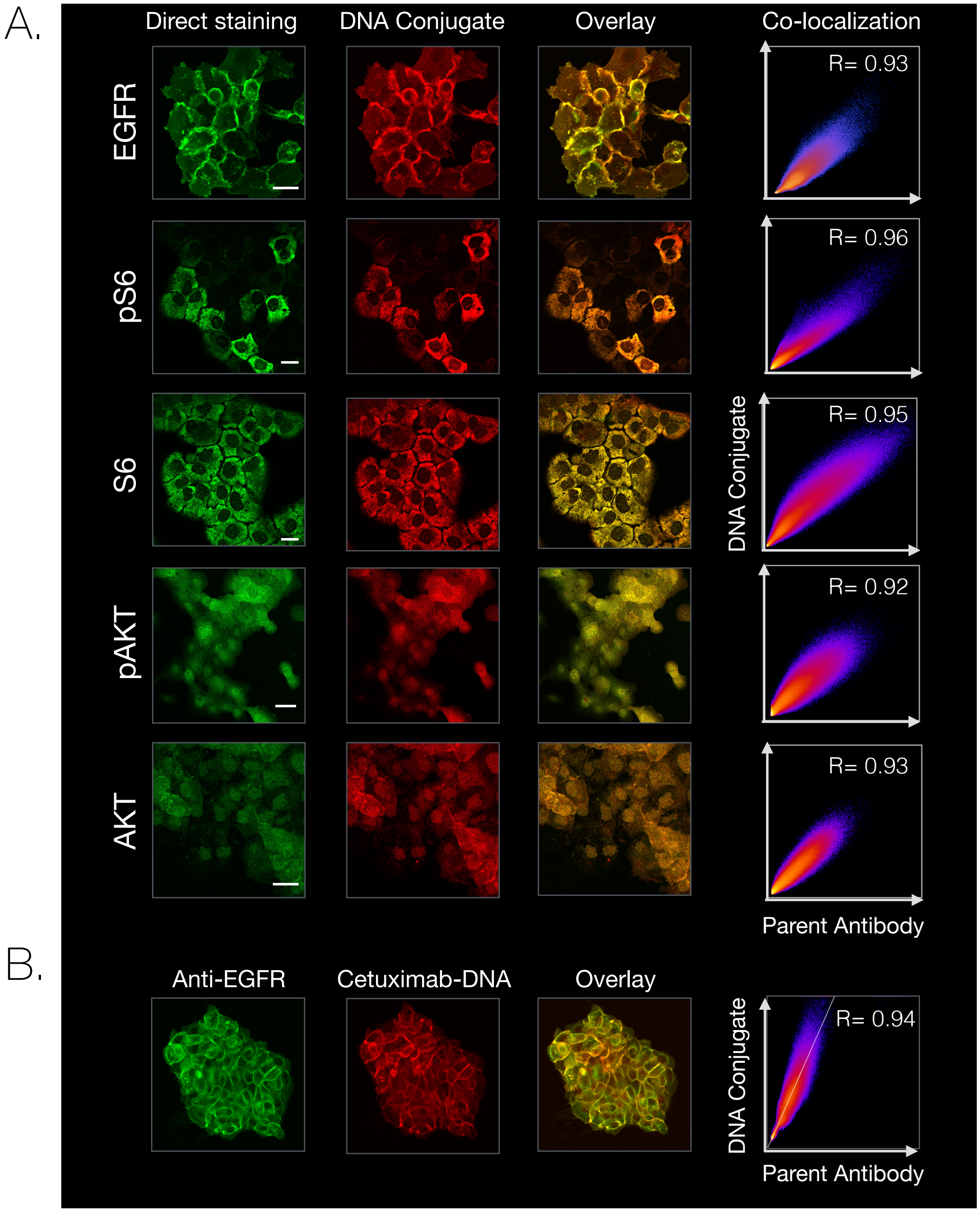

Supplementary Figure 3: Accurate target imaging with mAb-DNA. The mAb-DNA barcode imaging method is validated for five key targets in A431 cells by simultaneous staining. A. Cells were first treated with the unlabeled primary antibody and AF488 secondary antibody, followed by barcode imaging with mAb-DNA-AF647. Right column: the horizontal axis displays pixel intensity values for the direct staining image and the vertical axis describes intensity values for the DNA conjugate staining; Pearson's R value is shown for each target (overall mean = 0.94). Scale bars represent 50 μm. B. Cells were treated with an unlabeled sheep anti-EGFR primary antibody, washed, then stained with a donkey anti-sheep AF488 secondary antibody, followed by addition of cetuximab-DNA-AF647.

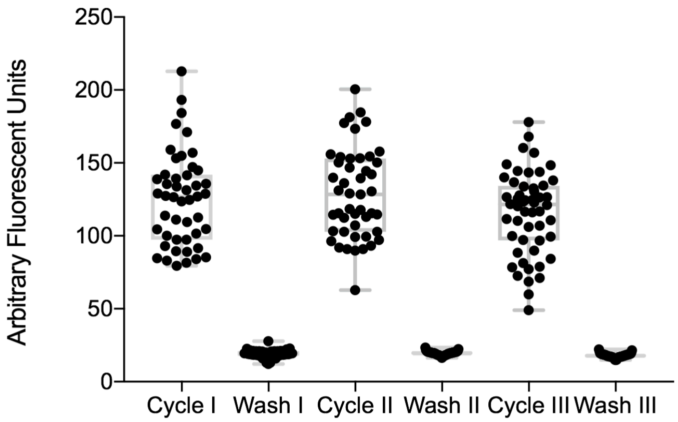

Supplementary Figure 4: Background Signal does not accumulate across cycles. Quantification of single-cell fluorescence intensity in A431 cells exposed to repeated treatments of Cetuximab-DNA-AF647 and washout conditions.

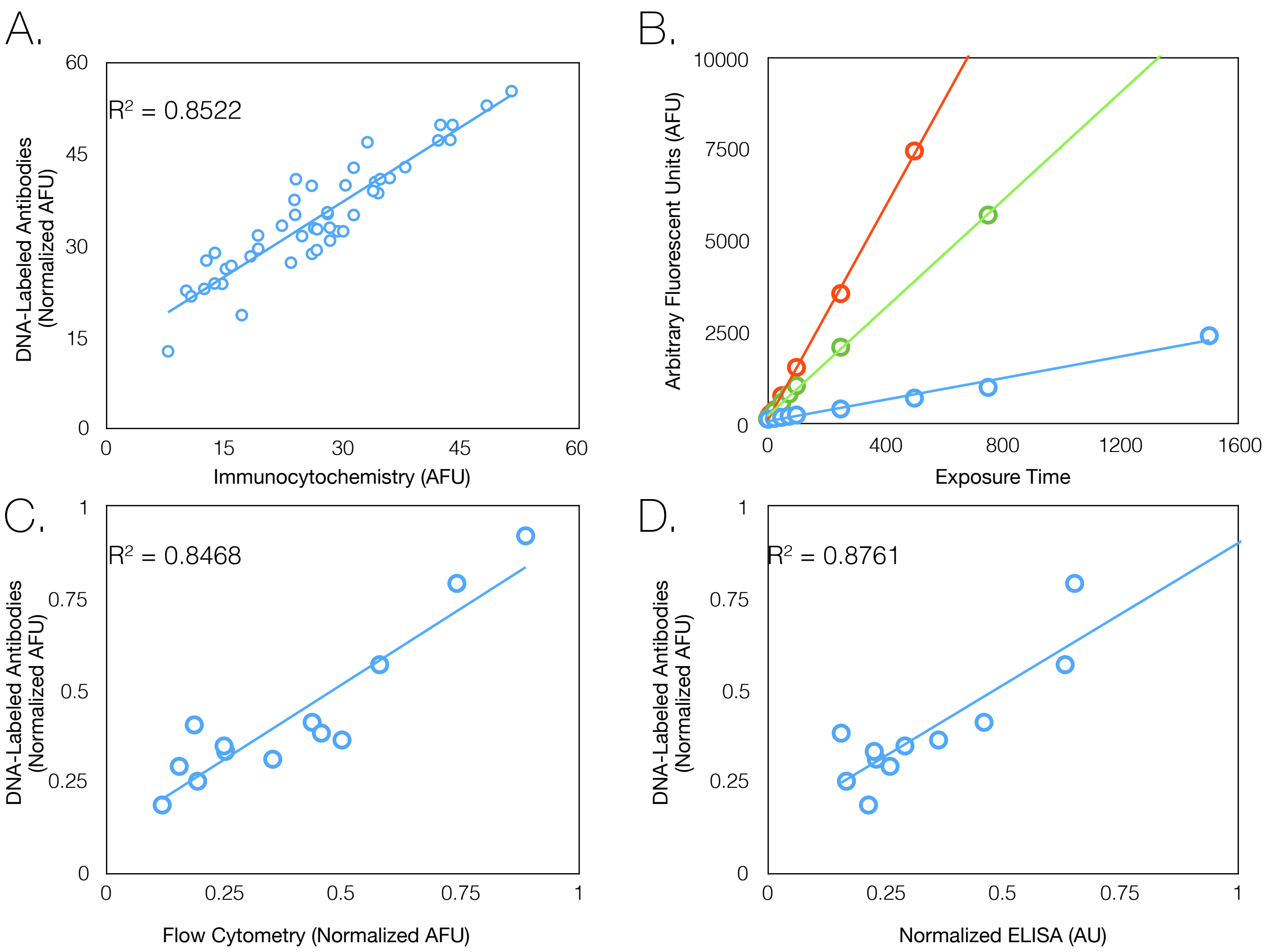

Supplementary Figure 5: Validation experiments. A. Scatter plot of normalized AFU values for single cell intensities of EGFR-labeled A431 cells as analyzed by mAb-DNA (y-axis) or immunocytochemistry (x-axis). **B.** Fluorescence intensity as a function of exposure for PacificBlue, AF488, and AF647 and their respective filter cube sets. **C.** Average fluorescence intensity values as calculated by SCANT for a set of protein and cell line targets as compared to Flow Cytometry (C) and ELISA (**D**).

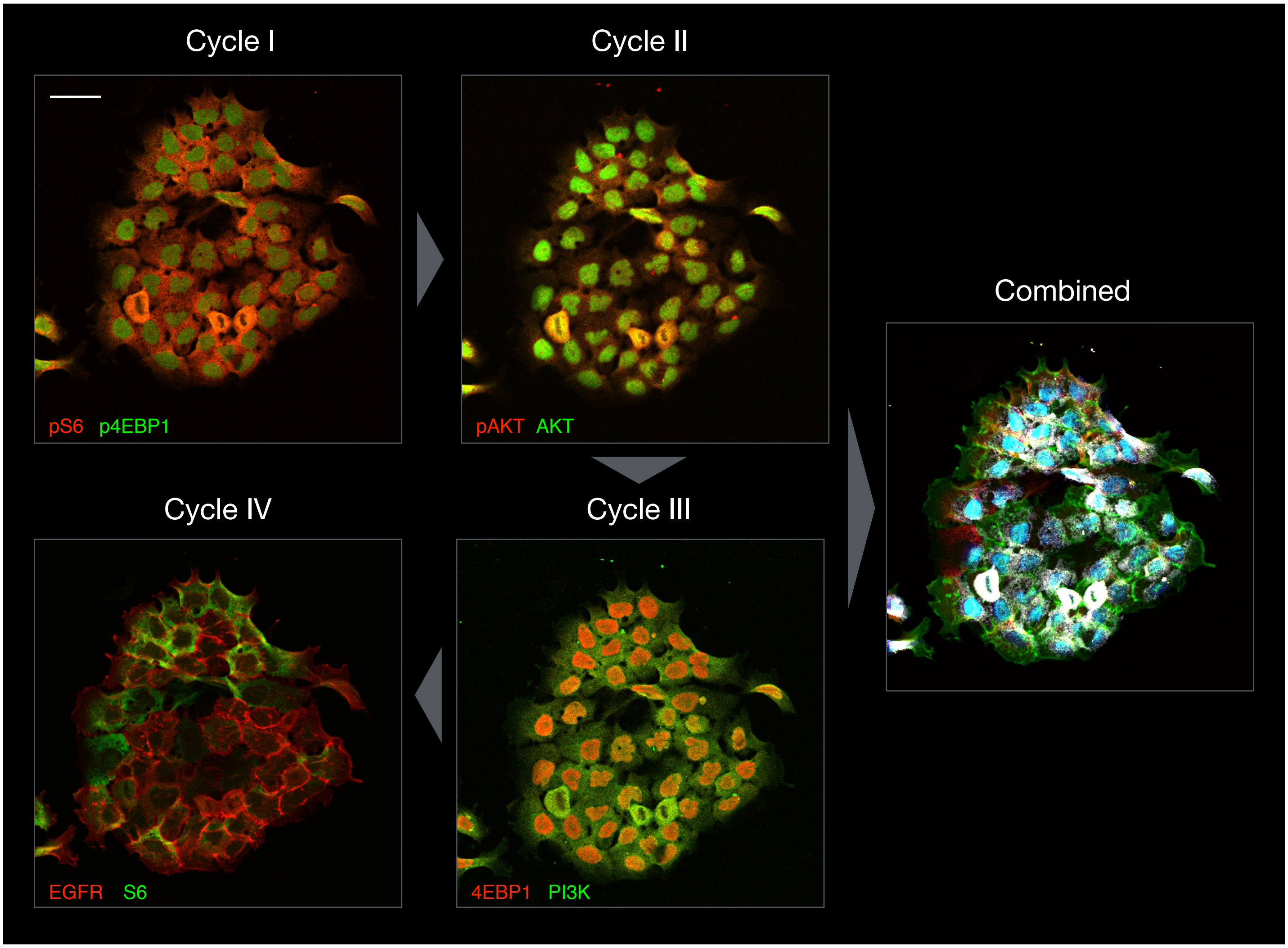

Supplementary Figure 6: Example of dual channel imaging and quaternary cycling. Dual channel imaging through 4 cycles in cultured A431cells reveals intracellular location of PI3K pathway markers (Cycle I: Red, pS6 and Green, p4EBP1; Cycle II: Red, AKT and Green pAKT; Cycle III: Red, 4EBP1 and Green, PI3K; Cycle IV: Red, EGFR and Green, S6). Scale bar (top left) represents 70 μm.

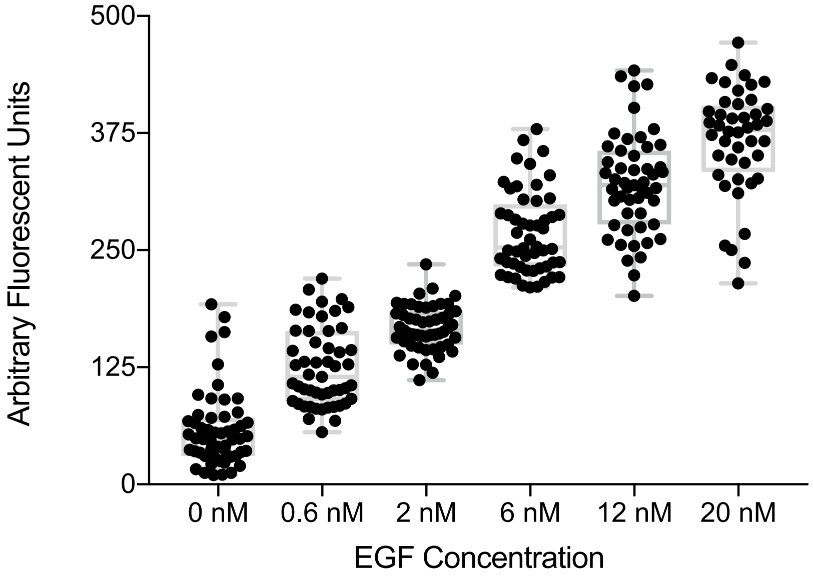

Supplementary Figure 7: Quantification of pS6 levels in EGF treated MCF-10A cells. MCF-10A cells were serum starved overnight and then exposed to varying concentrations of EGF. Quantification of pS6 in these cells illustrated the ability of a DNA conjugated antibody for pS6 to quantify a large range of pS6 levels.

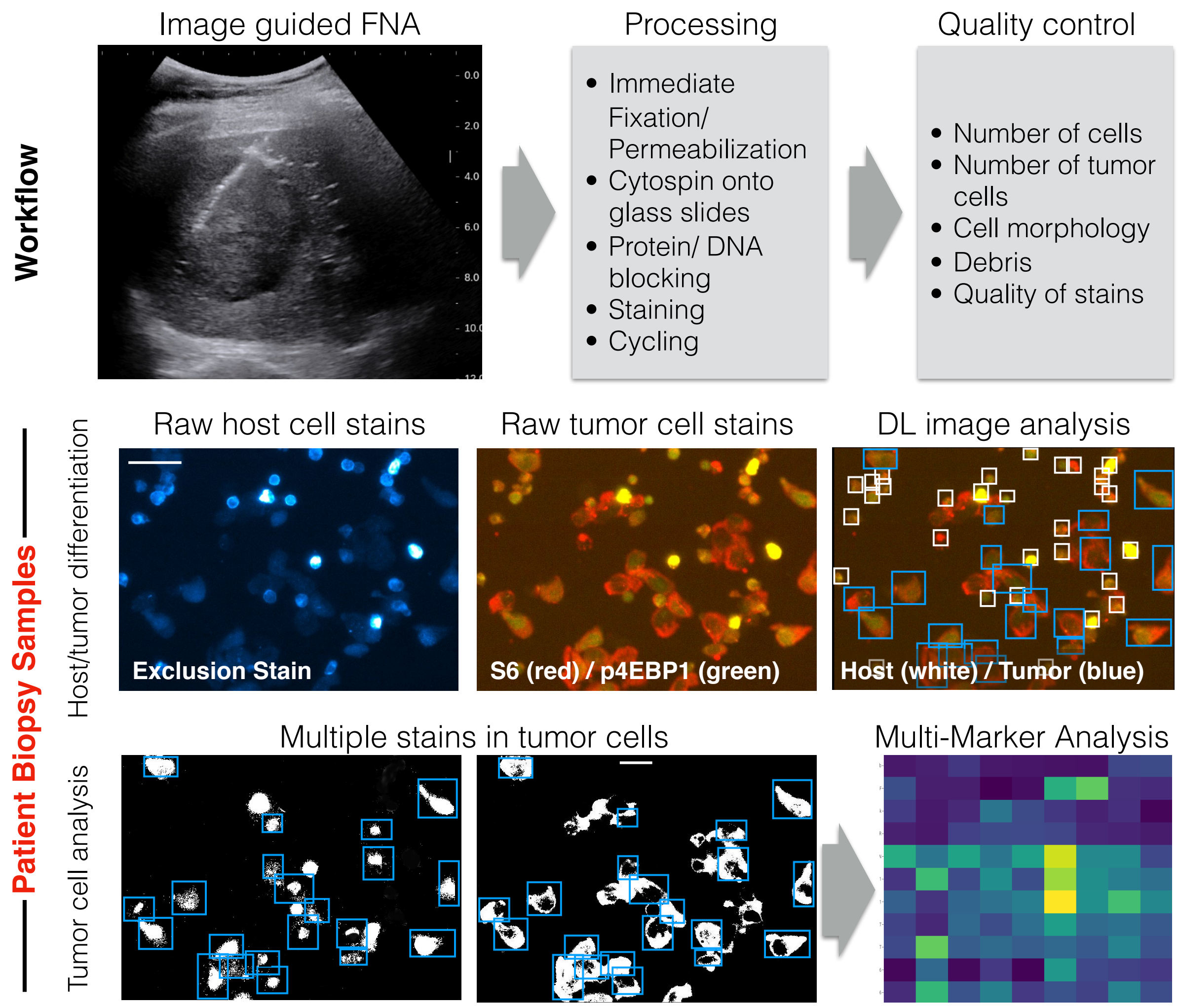

Supplementary Figure 8: Single cell analysis workflow for patient biopsy samples. FNA samples are first obtained under image guidance. Following acquisition, tumor cells are gently separated into single cell clusters and then fixed, spun onto glass slides and subjected to the SCANT staining protocol and cycling. Samples were quality controlled for downstream analysis by observations of total cell number, morphology, and cellular density on the slide. Following staining, a convolutional neural net based algorithm allows identification of both host cells and tumor cells, including their boundaries. Tumor cells that overlapped or resided too close to host cells were eliminated from analysis. Following segmentation of individual areas for each respective stain, the multi-marker analysis was calculated on normalized fluorescence values as described. Scale bar (top left) represents 80 μm.

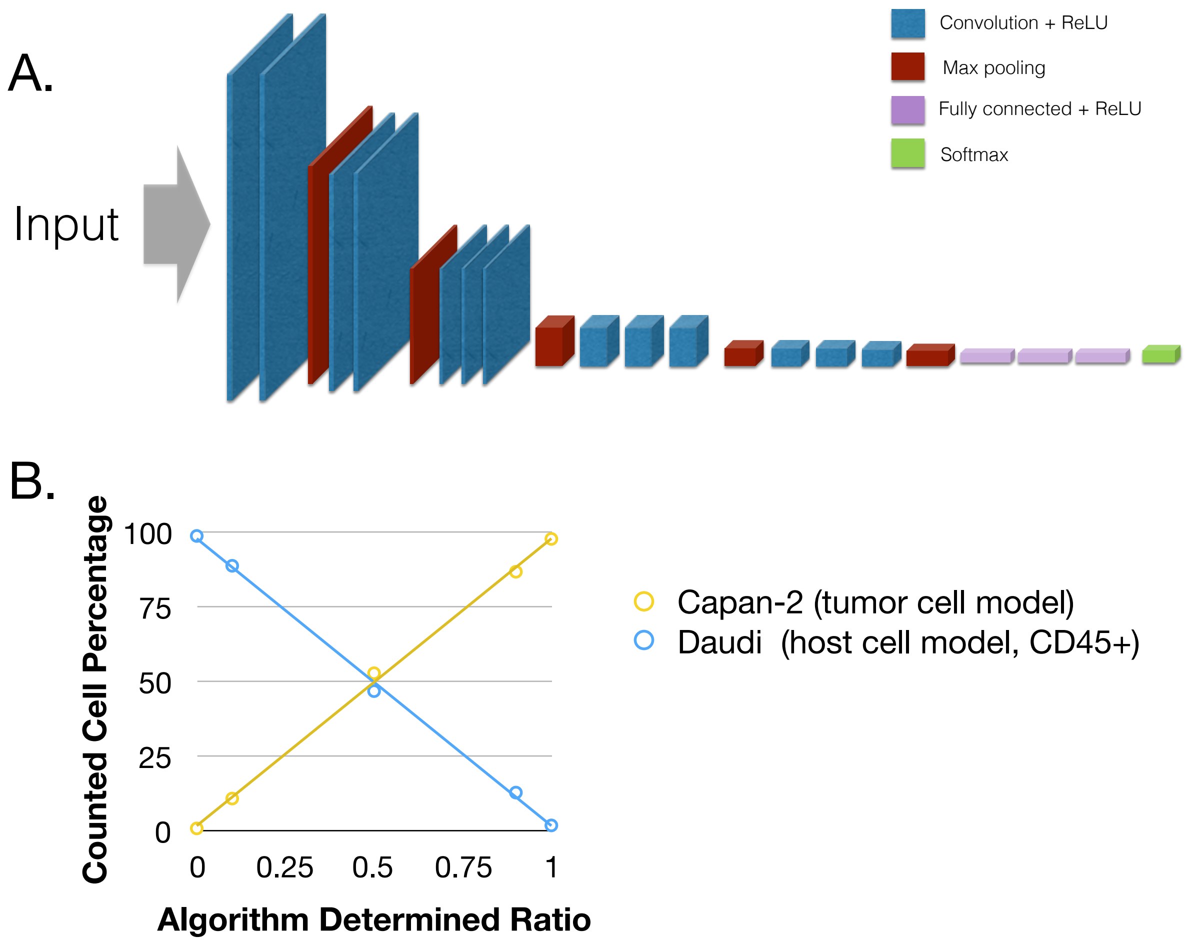

Supplementary Figure 9: CNN development and experimental validation. A. Detailed description of the VGG16 CNN, illustrating convolutional, max pooling, fully connected and softmax (classification) layers. B. To validate the algorithm, Capan-2 cells (simulating tumor cells) were combined with Daudi cells (CD45+ cells, simulating host cells) in varying ratios based on an initial count. Images of these varying ratios were taken and fed into the finalized CNN, where the ratios determined closely followed the expected proportions from the human counts used to generate the mixtures.

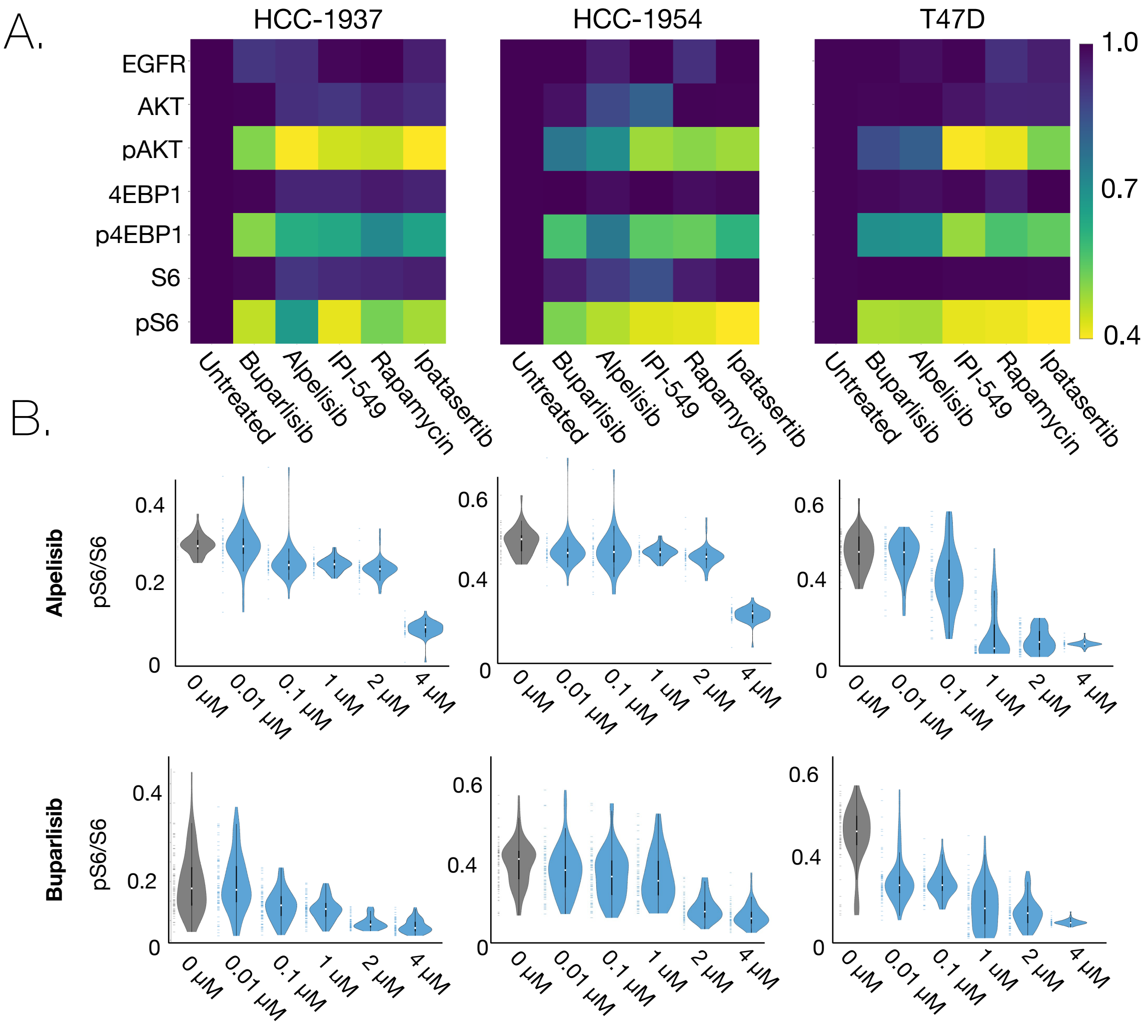

Supplementary Figure 10: Pathway analysis in different breast cancer cell lines with model drugs. A. Image analysis of ~1000 HCC1937, HCC-1954 and T47D breast cancer cells after exposure to targeted therapies. Note the decrease in pAKT, p4EBP1 and pS6 following treatment with the shown inhibitors. **B**. Dose response analysis of alpelisib (BYL-719) and buparlisib (BKM120), selective PI3K inhibitor of p110α and p110α/β/δ/γ respectively. The violin plots show sample heterogeneity to response and are consistent with a IC_{50} of <1 µM in the T47D cell lines.

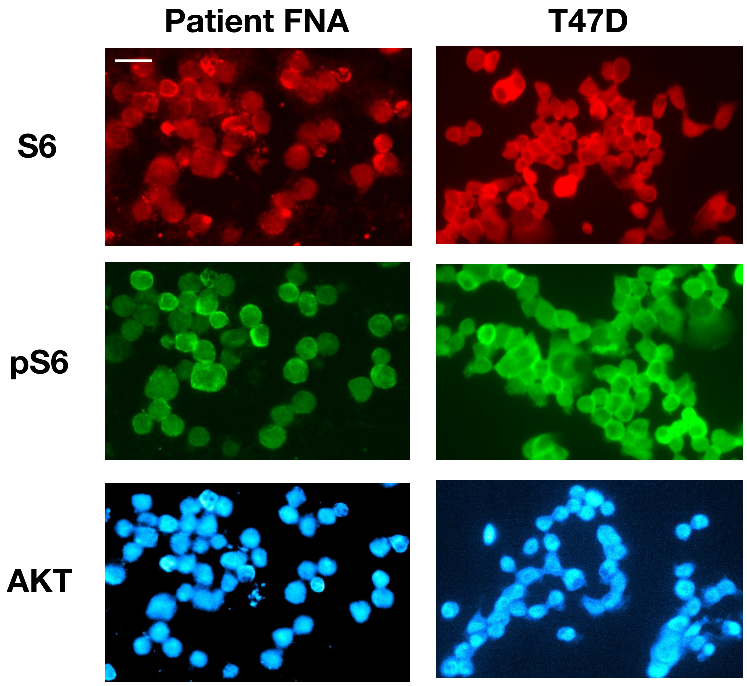

Supplementary Figure 11: Protein expression in patient samples and cell culture. Comparison of target staining between representative patient FNA cells and cultured T47D cells for a group of representative total and phospho targets. Scale bar (top left) represents 70 μm.

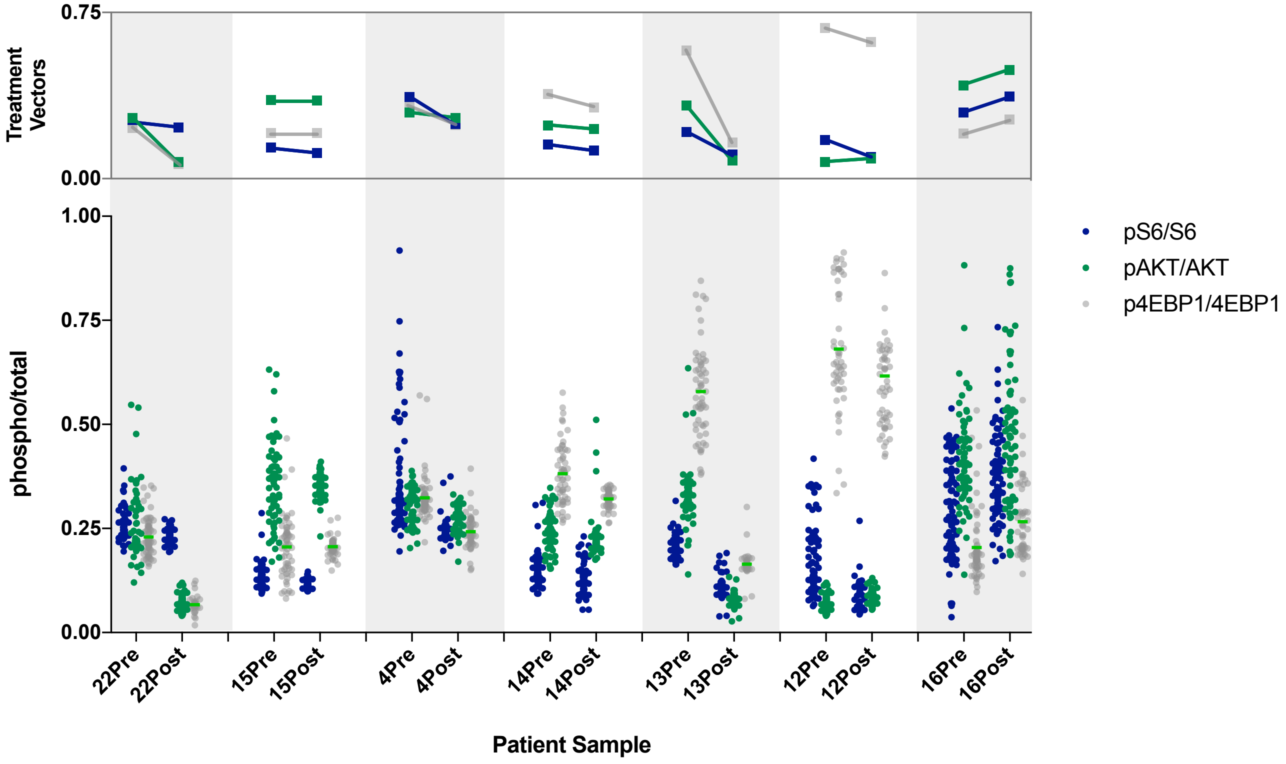

Supplementary Figure 12. All patient responses. Treatment vectors and single cell data for pS6/S6 (blue), pAKT/AKT (green) and p4EBP1/4EBP1 (gray) for all patients before and following treatment.

Supplementary Table 1: Antibodies. All antibodies used in this study including the average degree of labeling for each.

	Antibody	Host Species	Catalog	Vendor	Degree of Labeling
1	EGFR	Human/ Mouse	Cetuximab	Bristol-Myers Squibb	4.4
2	BRCA1 (A8X9F)	Rabbit	14823BF	Cell Signaling Technology	6.2
3	BRCA2	Rabbit	ab90541	Abcam	1.7
4	PI3 Kinase p110α (C73F8)	Rabbit	4249BF	Cell Signaling Technology	2.9
5	p-Akt (Ser473) (D9E)	Rabbit	4060BF	Cell Signaling Technology	3.2
6	total Akt (C67E7)	Rabbit	4691BF	Cell Signaling Technology	2.7
7	PTEN (217702)	Mouse	IC847T	Novus Biologicals	1.7
8	Phospho-S6 ribosomal protein (Ser240/244) (D68F8)	Rabbit	5364BF	Cell Signaling Technology	2.8
9	S6 Ribosomal protein (54D2)	Mouse	2317BF	Cell Signaling Technology	2.3
10	Phospho-4E-BP1 (Thr37/46) (236B4)	Rabbit	2855BF	Cell Signaling Technology	2.3
11	4EBP1 (53H11)	Rabbit	9644BF	Cell Signaling Technology	3.9
12	E2F1	Rabbit	ab112580	Abcam	1.9
13	Rb	Rabbit	ab181616	Abcam	2.3
14	Anti-alpha-smooth muscle actin	Rabbit	NB600-531	Novus Biolgicals	N/A
15	CD31 (C31/1395R)	Rabbit	NBP2-54385V	Novus Biologicals	N/A
16	CD45 (HI30)	Mouse	NBP1-79127V	Novus Biologicals	N/A

Supplementary Table 2: Oligos. Oligos utilized in this study including the mAb-barcode conjugated to all antibodies, imaging strands for each channel and capping/ blocking sequences.

	Sequence	Use	Vendor
**MAb-barcode**	5ThioMC6-D/ CAC TTC CAA AAC TTT TAA ACT ATT ATC ACA CCA AAT TCT ACT TAA TAC ACA ATA CAA CAC ACA	Labeling antibodies	Integrated DNA Technologies
**Imaging strand**	/5Alex488N/TGTGTGTTGTATT/3AlexF488N/	AF488 conjugate	Integrated DNA Technologies
	/5Alex594N/TGTGTGTTGTATT	AF594 conjugate	Integrated DNA Technologies
	/5Alex647N/TGTGTGTTGTATT/3AlexF647N/	AF647 conjugate	Integrated DNA Technologies
	/5AmMC6/TGTGTGTTGTATT/3AmMO/	Pacific blue conjugation	Integrated DNA Technologies; ThermoFisher Scientific
**Capping strand**	5'TGT GTG TTG TAT TGT GTA TTA AGT AGA ATT TGG TGT GAT AAT AGT 3'	Capping the primary barcode sites on antibodies from previous imaging cycles	Integrated DNA Technologies

Supplementary Table 3: Summary of staining conditions explored

Condition	Components	Performance	Comments
PBS	Typical Phosphate Buffered Saline	Poor	High Nuclear Staining
Commercial buffer	Odyssey Blocking buffer for Western blots (Licor)	Poor	High Nuclear Staining
CSB^{1}	Odyssey buffer, 1 mg/mL Salmon Sperm DNA, 25 uM of random sequence (24-mer), and 0.1% Triton-X 100	++	Reduced Nuclear Staining, increased cytosolic
CSB^{2}	Odyssey buffer, 1 mg/mL Salmon Sperm DNA and 0.1% Triton-X 100	++	High Nuclear Staining
CSB^{3}	Odyssey buffer, 1 mg/mL Salmon Sperm DNA and 0.1% Tween	+	No reduction in Nuclear Staining
CSB^{4}	Odyssey buffer, 1 mg/mL Salmon Sperm DNA, Triton-X 100 and 1 M NaCl	++	High ion concentration only has limited effect on nuclear staining
CSB^{5}	Odyssey buffer, 1 mg/mL Salmon Sperm DNA poly-T-blocker, random sequence 24mer blocker and 0.1% Triton-X	++++	Similar effect to CSBx buffer below, additional cost not justified
CSB^{x}	Odyssey buffer, 1 mg/mL Salmon Sperm DNA, 25 uM of poly-T blocker (24-mer), and 0.1% Triton-X 100	++++	Works for all
